# Performance and Stability of Corundum‐type In_2_O_3_ Catalyst for Carbon Dioxide Hydrogenation to Methanol

**DOI:** 10.1002/anie.202416990

**Published:** 2025-01-10

**Authors:** Albert Gili, Georg Brösigke, Mudassar Javed, Emiliano Dal Molin, Philipp Isbrücker, Jens‐Uwe Repke, Franziska Hess, Aleksander Gurlo, Reinhard Schomäcker, Maged F. Bekheet

**Affiliations:** ^1^ Technische Universität Berlin Faculty II Mathematik und Naturwissenschaften, Institut für Chemie Straße des 17. Juni 135 10623 Berlin Germany; ^2^ Helmholtz-Zentrum Berlin für Materialien und Energie 14109 Berlin Germany; ^3^ Technische Universität Berlin Process Dynamics and Operations Group, KWT9 Straße des 17. Juni 135 10623 Berlin Germany; ^4^ Technische Universität Berlin Faculty III Process Sciences, Institute of Materials Science and Technology, Chair of Advanced Ceramic Materials Straße des 17. Juni 135 10623 Berlin Germany

**Keywords:** Heterogeneous catalysis, synchrotron in situ XRD, carbon dioxide activation, structure–activity correlations, oxygen vacancy, DFT

## Abstract

Carbon dioxide hydrogenation to methanol is a key chemical reaction to store energy in chemical bonds, using carbon dioxide as an energy sink. Indium oxide is amongst the most promising candidates for replacing the copper and zinc oxide catalyst, which is industrially applied for syngas mixtures but less idoneous for educts with carbon dioxide due to instability reasons. The polymorph of indium oxide and the operating conditions remain to be optimized for optimal and stable performance. Indium oxide catalysts containing different rhombohedral and cubic phase ratios were synthesized using a solvothermal method and evaluated in an ideally mixed gas phase reactor. The pure rhombohedral catalyst shows the best performance in terms of yield to methanol, selectivity, and stability. The phase stability was assessed using synchrotron in situ XRD and Rietveld refinement, shedding light on the stability of the different phases at extended operating conditions. Depending on the flow rate, temperature, and hydrogen partial pressure, a rhombohedral to cubic transition occurs, ultimately yielding inactive metallic indium. If present, cubic In_2_O_3_ serves as nuclei to induce the cubic to rhombohedral transition, hampering performance. These results allow for a more rational catalyst design and fine‐tuning of the operating conditions to ensure optimal and stable performance.

## Graphical abstract

The rhombohedral polymorph of indium oxide performs better than the cubic one in terms of yield, selectivity, and stability for the carbon dioxide hydrogenation to methanol. The catalyst stability and sequence of transformation from rhombohedral to cubic to metallic indium is revealed in situ using synchrotron X‐ray diffraction.

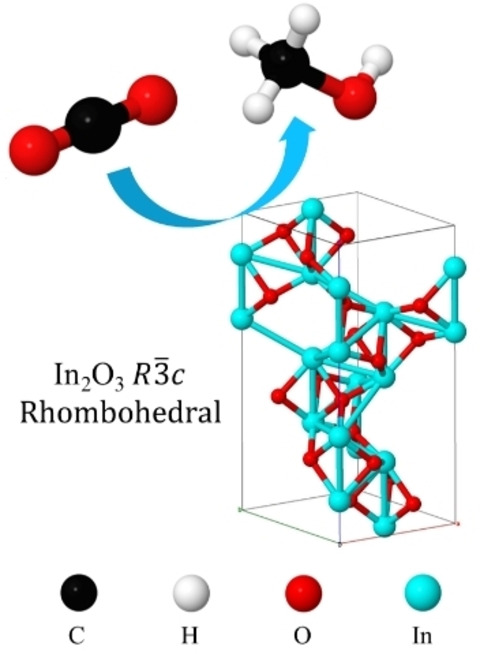

## Introduction

The heterogeneous catalytic activation of CO_2_ to fuels, including methanol (Reaction 1), simultaneously tackles two of the greatest challenges that modern society faces: anthropogenic greenhouse gas emissions and fossil fuel depletion.^[1]^ In the context of the desired energy transition, methanol plays a key role:^[2]^ it is an excellent fuel, a key building block for several industrial reactions, and can directly be used in solid‐oxide fuel cells (SOFC).^[3]^ The storage of electricity produced with renewable sources in the form of chemical bonds by using CO_2_ as an energy sink is a promising concept that allows the integration of renewable sources into the existing electric grid. The state‐of‐the‐art industrially applied catalyst for methanol synthesis from mixed syngas (CO/CO_2_/H_2_) is the Cu−ZnO/Al_2_O_3_ catalyst. Nevertheless, it suffers from drawbacks when applied to CO_2_ hydrogenation, mainly by limited reaction rates, low selectivity (due to the Cu‐catalyzed reverse water‐gas shift reaction^[4]^ (RWGS‐Reaction 2)), and decreased stability.^[5]^


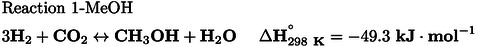




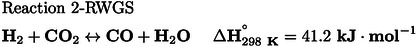




In the search for more selective and stable catalysts for CO_2_ hydrogenation to methanol, cubic bixbyite‐type c‐In_2_O_3_ (space group *Ia*‐3, No. 206) was first theoretically suggested by Ye et al.^[6]^ and experimentally demonstrated by Martin et al.^[5]^ The later work proved the high yields, impressive selectivity, and outstanding stability of the c‐In_2_O_3_ and c‐In_2_O_3_/ZrO_2_ catalysts. Oxygen vacancies (V_O_) were proposed to be the active site for the reaction over pure c‐In_2_O_3_, and the use of ZrO_2_ as support increased the concentration of vacancies as well as stabilized the catalytic performance. Perez‐Ramirez's group would further investigate In_2_O_3_‐based catalysts for this reaction,[^7^, ^8^, ^9^] including atomic‐scale engineering with Pd to boost rates^[10]^ and feed optimization.^[11]^ In_2_O_3_ is known to crystallize in other metastable polymorphs such as rhombohedral corundum‐type (*R*‐3*c*, No. 167, rh‐In_2_O_3_) and orthorhombic (*Pbcn*, No. 60, o‐In_2_O_3_).[^12^, ^13^, ^14^] Recently, rh‐In_2_O_3_ has been theoretically and experimentally studied by Dang et al.,^[15]^ with the rh‐In_2_O_3_ with (104) exposed facet being superior in terms of space‐time yield (STY), selectivity, and high stability.^[15]^ Furthermore, mixed‐phase In_2_O_3_ catalysts containing both c‐ and rh‐polymorphs were evaluated, resulting in improved performance compared to the single‐phase catalysts, with an optimal cubic phase content of 20.6 %.^[16]^ Simultaneous operando synchrotron‐based X‐ray diffraction (XRD) and X‐ray absorption fine structure (XAFS) study on c‐In_2_O_3_ catalyst for CO_2_ hydrogenation (300 °C, 20 bar and H_2_ : CO_2_ : N_2_=3 : 1 : 1) identified three distinct performance phases that correlate to changes in the catalyst structure.^[17]^ Firstly, an activation phase with increasing STY_MeOH_ resulting from an increase in the concentration of V_O_ in c‐In_2_O_3_ proved by the decrease of the In−O coordination number/partial reduction of In; a second stable phase with a relatively stable structure of the catalyst, which leads to a third phase of deactivation characterized by an amorphization of the cubic crystal leading to molten In^0^. Supporting c‐In_2_O_3_ on monoclinic ZrO_2_ resulted in the formation of a ZrO_2_ : In solid solution with V_O_ being the active site in the atomic array In−V_O_−Zr.^[18]^ This configuration prevents the over‐reduction of c‐In_2_O_3_ into its metallic form and maximizes yield and stability.^[18]^ The facet‐dependency on the activation barriers has been modeled using DFT on the c‐In_2_O_3_(110),[^6^, ^15^, ^19^, ^20^, ^21^] c‐In_2_O_3_(111),[^6^, ^15^, ^19^, ^20^] rh‐In_2_O_3_(012),^[15]^ and rh‐In_2_O_3_(104),^[15]^ with the latest predicted to be the best performing surface. However, a detailed understanding of the structure‐performance correlation for different In_2_O_3_ polymorphs and their stability during reaction for catalyzing CO_2_ hydrogenation to methanol is currently lacking.

In this work, we have synthesized In_2_O_3_ catalysts containing different ratios of rhombohedral and cubic phase using a solvothermal method, as determined by refinement of the XRD data. These samples have been investigated for CO_2_ hydrogenation to methanol in a continuously stirred tank reactor (CSTR)/gradient‐less reactor and evaluated in terms of the STY to methanol, CO_2_ reaction rate, and selectivity toward methanol. The phase stability during the reaction was assessed using synchrotron in situ XRD, followed by Rietveld refinement of the XRD data combined with DFT, which shed light on the stability of the different phases. The results allow for a more rational catalyst design and fine‐tuning of the operating conditions to ensure optimal and stable performance.

## Results and Discussion

**Figure 1:**
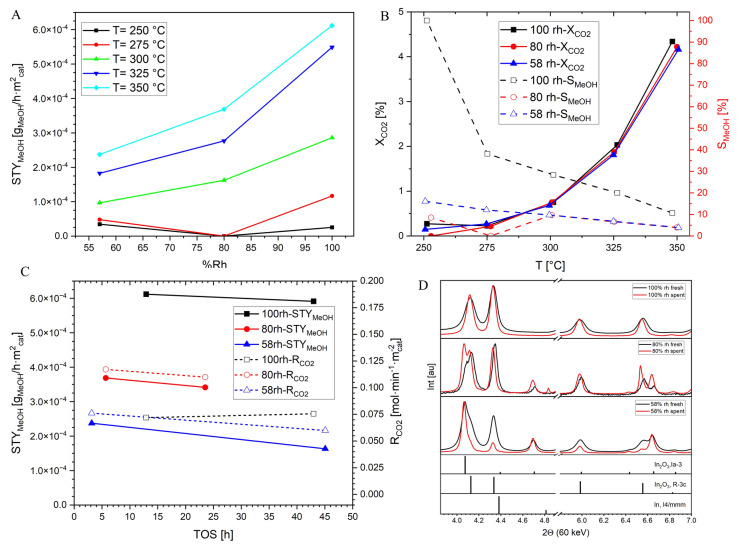
Panel A: STY_MeOH_ as a function of the wt.% of rh‐In_2_O_3_ phase at different T's. Panel B: X_CO2_ and S_MeOH_ as a function of temperature. Panel C: STY_MeOH_ and R_CO2_ for the different samples with TOS at 350 °C. Conditions: P=20 bar, WHSV=20000 NmL ⋅ h^−1^ ⋅ g_cat_^−1^, CO_2_ : H_2_ : N_2_=1 : 3 : 1 [−]. Lines are added for easier visualization. Panel D: pre‐ and post‐catalytic ex situ XRD data of the 3 samples. The intensity has been normalized, and the experiment performed with Cu_kα1_ radiation has been transformed to 60 keV.

The phase composition and specific surface area (SSA) of the as‐prepared catalysts were characterized by ex situ XRD and nitrogen sorption analysis. As depicted in Figure 1‐D, all observed XRD reflections can be indexed to c‐In_2_O_3_ (PDF #00‐006‐0416) and rh‐In_2_O_3_ (PDF #00‐022‐0336). Rietveld refinement (RR) of the XRD data reveals that the weight fractions (wt.%) of the rh‐In_2_O_3_ phase are 100 wt %, 80 wt %, and 58 wt %, respectively (Table SI1). The formation of c‐In_2_O_3_ in the samples is accompanied by an increase in the average crystallite size (CS) of both rh‐In_2_O_3_ and c‐In_2_O_3_ phases and, consequently, a slight decrease in the SSA of the samples. The performance indicators have been normalized to the SSA.

Figure 1‐A shows the STY_MeOH_ of the catalysts as a function of the wt.% of rh‐phase at T=250–350 °C and 20 bar. For all the samples, the yields increase with temperature increases of up to 350 °C. An increase in the wt.% of the rh‐phase results in higher yield (except for the lower temperatures of the 80 %rh‐sample): the bare rh‐In_2_O_3_ catalyst performs the best at all the conditions tested. Comparison of the best performing 100 %rh‐In_2_O_3_ with the commercial Cu/ZnO/Al_2_O_3_ (CZA) catalyst is made to allow comparison with literature (see Figure SI4): our system yields higher STY of MeOH above T=300 °C due to an improved selectivity. Similar trends were already reported.^[5]^ Figure 1B shows the conversion of CO_2_ and selectivity toward MeOH as a function of temperature for the three samples: an increase in temperature results in an increase of R_CO2_, mainly via RWGS (Reaction 2, see the system's ΔG_i_° in Figure SI2 and $S_{CO}$ in Figure SI3). $S_{MeOH}$ is particularly high for the 100 %‐rh sample at 250 °C, reaching 100 %. Similar trends of improved S_MeOH_ in the rh‐polymorph were already observed.^[15]^ S_MeOH_ and R_CO2_ can be further enhanced by increasing the H_2_ : CO_2_ ratio^[15]^ or by increasing the pressure.[^5^, ^22^] Interestingly, the 80 %rh sample shows the highest R_CO2_ of all samples (see Figure 1C), the same ratio as previously reported in another study.^[16]^ Nevertheless, the best performance in terms of STY_MeOH_ is achieved by the pure rh‐In_2_O_3_ and not by a mixed phase In_2_O_3_. To allow comparison with the literature, the best STY shown for the 100 %rh‐In_2_O_3_ at 350 °C corresponds to 0.025 kg_MeOH_ ⋅ kg_cat_^−1^ ⋅ h^−1^. The stability of the samples with TOS is shown in Figure 1C (the first and last data points at the same temperature are taken from a full series of experiments at different temperatures). For all samples, the STY_MeOH_ slightly decreases with TOS, basically following the decrease in R_CO2_, except for the 100 %‐rh sample, for which a constant X_CO2_ with decreasing STY_MeOH_ is observed.

Pre‐ and post‐catalysis XRD analysis in Figure 1D shows the stability of the rh‐phase in the 100 %rh catalyst, whereas no phase transformation takes place, and only an increase in the CS from 9.5±1 to 13.9±1 nm is observed (Table SI1, obtained from Rietveld refinement). In contrast, the already present nanocrystalline c‐In_2_O_3_ phase in the 80 %rh and 58 %rh samples promotes the rh‐In_2_O_3_→c‐In_2_O_3_ transition, decreasing the wt.% of rh‐In_2_O_3_ from 80 %rh to 62 wt % and 58 %rh to 20 wt %, respectively, resulting as well in a stronger increase of the crystallite size of both polymorphs (Table SI1). The loss of surface area resulting from crystallite growth can explain the change in the R_CO2_ in Figure 1C: hindered‐growth 100 %rh‐In_2_O_3_ maintains the rates while growing rh and c in the mixed oxides reduces the reaction rates. The metastability of the rh‐In_2_O_3_ polymorph and a kinetically enhanced transition rh→c for those systems that already include cubic nuclei explain the differences observed.

**Figure 2:**
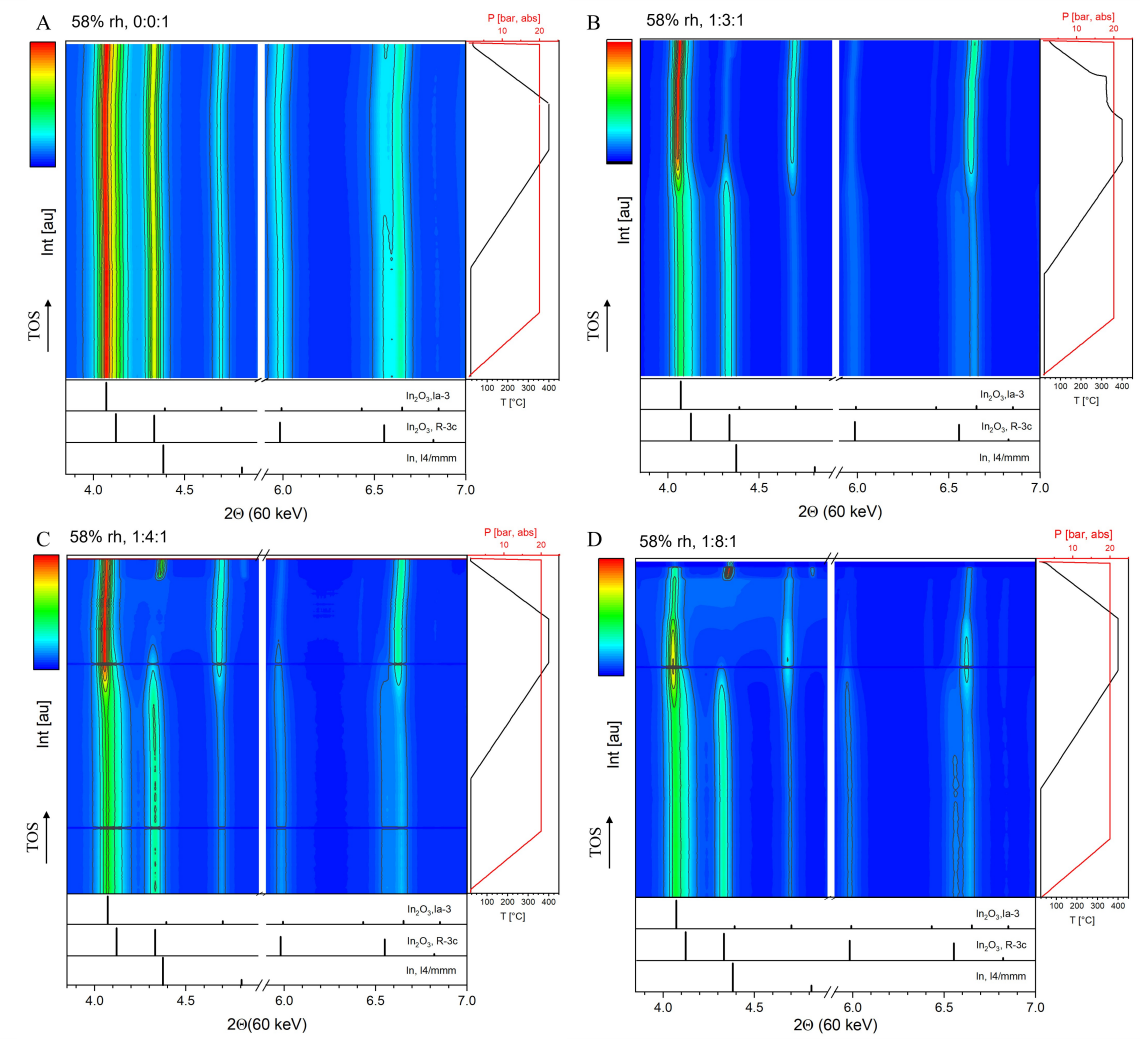
In situ XRD data of the 58 %rh‐In_2_O_3_ sample under (A) pure argon (B) CO_2_ : H_2_ : Ar=1 : 3 : 1, (C) CO_2_ : H_2_ : Ar=1 : 4 : 1, (D) CO_2_ : H_2_ : Ar=1 : 8 : 1 ratios. Conditions: P=1–20 bar_(abs)_, total flow rate of 5 NmL ⋅ min^−1^ for reaction (WHSV~200000 NmL ⋅ h^−1^ ⋅ g_cat_^−1^) and 25 NmL ⋅ min^−1^for the pressurizing step at RT; heating and cooling rates are 10 and 20 °C ⋅ min^−1^, respectively. Both hold steps extend over 15 min. Reference PDFs are In (I4/mmm, 00–005–0642), In_2_O_3_ (R‐3c, 00–022–0336) and In_2_O_3_ (Ia‐3, 00–006–0416).

**Figure 3:**
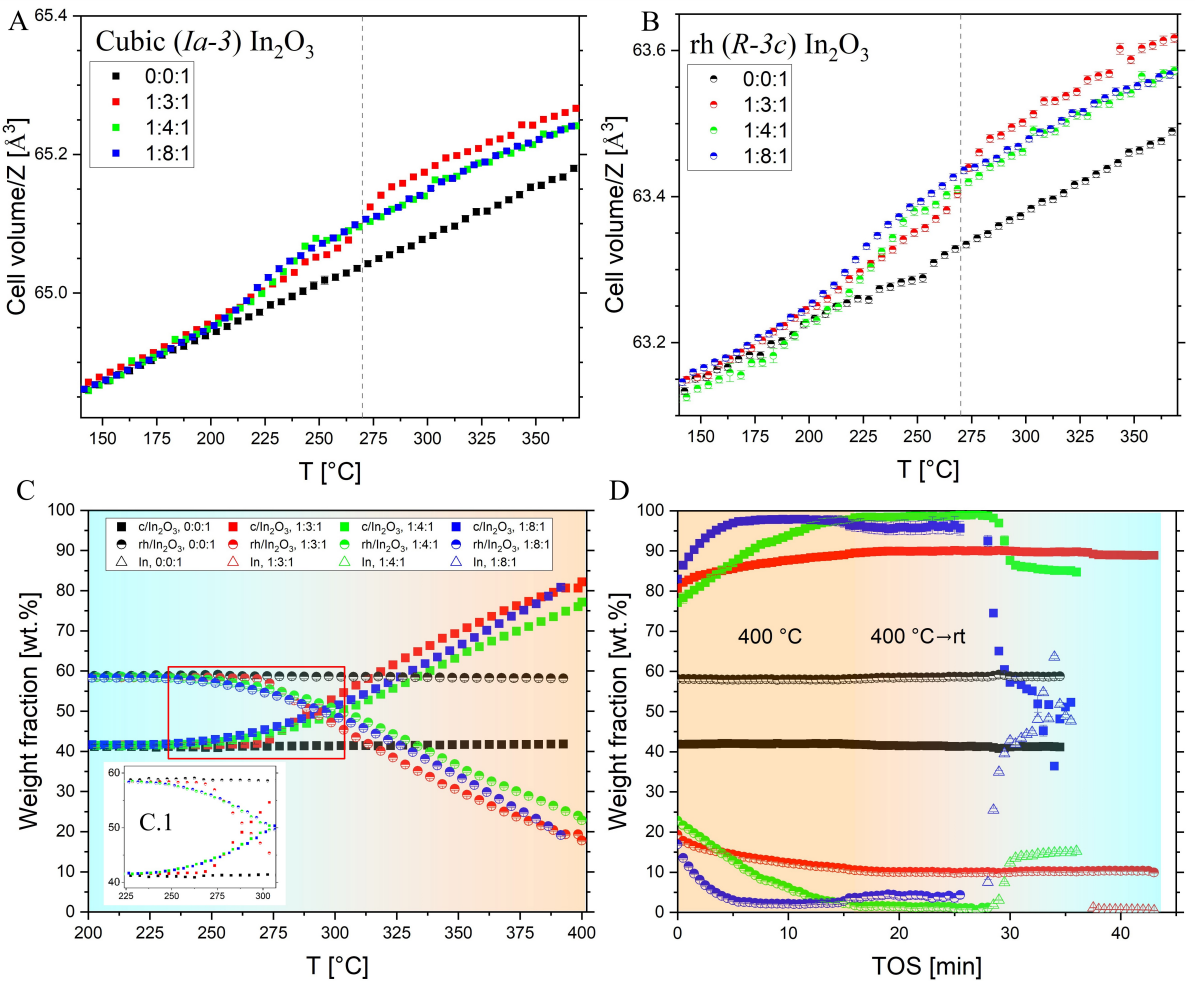
Cell volume/Z for the cubic (Panel A) and rhombohedral (panel B) polymorphs of the 58 %rh/In_2_O_3_ sample as a function of the temperature obtained by Rietveld refinement of the in situ data shown in Figure 2. Conditions: P=20 bar_(abs)_, total flow rate of 5 NmL ⋅ min^−1^ for reaction (WHSV~200000 NmL ⋅ h^−1^ ⋅ g_cat_^−1^) heating rate is 10 °C ⋅ min^−1^. Panel C: weight fraction of c/In_2_O3, rh/In_2_O_3,_ and In obtained as a function of the T, showing the onset of phase‐transitions (C.1). Panel D: continuation of panel E at 400 °C with TOS and later cooling to rt (symbols are the same as Panel C). TOS=0 is the moment the cell reaches 400 °C. Error bars are displayed for all data points.

Over‐reduction of In_2_O_3_ imposes a challenge for the stability of this catalyst, and should be hindered.^[17]^ A recent study^[16]^ revealed the stability of both c‐In_2_O_3_ and rh‐In_2_O_3_, as well as their phase mixtures, under catalytic conditions (P=30 bar, WHSV=7500 NmL ⋅ h^−1^ ⋅ g_cat_^−1^, CO_2_ : H_2_ : N_2_=1 : 3 : 1 and T=300 °C) different from our study. Improved performance in the mixed‐phase catalysts was observed compared to the single‐phase samples, with high stability in all the samples at the conditions tested but without in situ characterization. Thus, we have performed in situ synchrotron XRD to identify the structural changes during catalysis at extended reaction conditions and monitor the over‐reduction and its sequence toward the formation of metallic indium (In^0^). Figure 2 displays the phase stability of the 58 %rh‐In_2_O_3_ catalyst under relevant H_2_ : CO_2_ : Ar mixtures for CO_2_ hydrogenation to methanol up to 400 °C. The color‐contoured maps show the intensity of the crystallographic reflections (Y‐axis shows patterns following TOS from bottom to top) at the selected 2Θ range (X‐axis). The calculated reference patterns are shown, allowing us to identify c‐In_2_O_3_ (*Ia*‐3), rh‐In_2_O_3_ (*R*‐3*c*), and metallic In (*I*4/*mmm*). The cell was first pressurized under high flow but at room temperature, minimizing the undesired effects of high flow at elevated temperature, which can induce faster reduction. The Rietveld refinement results are shown in Figure 3. Under pure Ar atmosphere, no significant structural changes are observed, and the relative ratios of the two polymorphs remain constant (Figure 2A). The presence of H_2_ in the gas mixtures induces the rh→c phase transition and the appearance of metallic In (final content increases with increasing P_H2_), which can be observed upon solidification during cooling (T_f_~157 °C). The onset temperature of the rh→c transition decreases with an increase of the H_2_ ratio from 1 : 3 : 1 (~270 °C) to 1 : 4 : 1 (~230 °C), and remains constant for the 1 : 8 : 1 experiment. Interestingly, the rh→c transition is faster for the 1 : 3 : 1 experiment (see Figure 3C.1). During the isothermal step at 400 °C (Figure 3D), the transition continues. A higher P_H2_ results in faster depletion of the rh‐phase, which completely disappears for the 1 : 4 : 1 and 1 : 8 : 1 ratios. These results agree with the 58 %rh sample during catalysis (Figure 1D): the stable polymorph ratio in the 100 %rh sample shown in Figure 1D suggests that the presence of c‐In_2_O_3_ phase tends to destabilize the rh‐polymorph at these catalytic conditions, as previously stated. Another factor contributing to accelerated phase change could be the difference in flow rate between the catalytic tests and the in situ experiments (due to the setup's limitations). Previous work^[16]^ reported no change of the different polymorphs in mixed rh/c (rh wt.% of 73, 80 and 84) catalysts after reaction at 300 °C, 30 bar, 1 : 3 : 1 and 7500 NmL ⋅ h^−1^ ⋅ g_cat_^−1^.

Figure 3A and B show the cell volume normalized to the number of formula (In_2_O_3_) per unit cell Z for both polymorphs of the 58 %rh/In_2_O_3_ sample during the heating step of the in situ experiment shown in Figure 2. A relatively constant volume expansion occurs during the heating step (see Figure SI6), which is caused by thermal expansion. The absence of H_2_ in the gas phase (0 : 0 : 1) experiment results in no additional expansion of the unit cell induced by the generation of V_O_. For the H_2_‐containing experiments, an additional expansion of the lattice occurs: for the 1 : 4 : 1 and 1 : 8 : 1 experiments, V_O_ is generated^[23]^ at an onset temperature of ~220 °C. For the 1 : 3 : 1 experiment, this onset is slightly delayed to T~270 °C, though being the expansion more notorious. The onset of the generation of the V_O_ is the same for both polymorphs. After the onset and until 400 °C, no extra expansion or contraction besides pure thermal expansion can be seen. Figure SI5 displays the change in the crystallite size obtained from refining the XRD data shown in Figure 2. At 260 °C, the crystallite size of all the H_2_‐containing experiments starts to increase for both polymorphs. Interestingly, no growth in the crystallite size is observed during heating in 0 : 0 : 1 experiment, highlighting the importance of the chemical environment and catalyst activity in the growth process. For the 1 : 3 : 1 experiment, the crystallite size of both c‐In_2_O_3_ and rh‐In_2_O_3_ phases increases to about 16.5±1 nm at 350 °C, which is slightly lower than values determined for both phases in the spent catalyst at the same catalytic condition but after 45 h (Table SI1). This suggests that no significant crystallite growth of either phase occurs under isothermal conditions at 350 °C. A lower growth rate and smaller crystallite size of the rh polymorph are observed when the H_2_ ratio in the gas mixture increases, which can be explained by the fast phase transition of this phase into c‐In_2_O_3_ with an increasing H_2_ ratio. Using DFT calculations, we calculated the V_O_ formation energies (Figure SI7), the cell volume change upon the introduction of V_O_ (Figure SI8), as well as formation energies for both In_2_O_3_ polymorphs as a function of oxygen vacancy concentration (Figure SI9). The cell volume expands in both polymorphs up to a V_O_ concentration of 0.7 % (rh) and 2.1 % (c), at which maximum cell expansions of 0.17 % (rh) and 0.9 % (c) are reached. We note that the experimental cell volume expansions for both polymorphs are fairly similar to each other, at 0.15 % and 0.12 % for the rh‐ and c‐polymorphs. The V_O_ formation energy (Figure SI7, measured at [V_O_]=1 %) due to the reduction with H_2_ is slightly higher in the rh structure at 1.14 eV compared to 1.01 eV for the c‐polymorph, supporting the proposition that both materials contain significant amounts of V_O_ under the given conditions, which will result in a lattice expansion. However, under the given reduction conditions, In_2_O_3_ is not stable against reduction to In; hence, the observed V_O_ concentrations and structures do not reflect thermodynamic equilibrium. As shown in Figure S19, the difference between the formation energies of defective c‐In_2_O_3_ and rh‐In_2_O_3_ polymorphs as a function of the concentration of V_O_ is remarkably constant, and the cubic polymorph is more stable than the rhombohedral by 65.7 meV on average per InO_x_ unit. From this difference and the small entropy difference between the two polymorphs (4.2 J ⋅ K^−1^ ⋅ mol^−1^, see methods description in the ESI), we expect that the lattice energy is the decisive factor governing the relative stabilities of the c‐ and rh‐polymorphs in the temperature range studied here. However, as shown in Table SI1, the small crystallite size of rh‐In_2_O_3_ in the spent 100 %rh catalyst suggests that the size effect, which influences the surface energy and/or surface stress, might be the reason for the increase in the stability of the rhombohedral polymorph.[^24^, ^25^] Furthermore, the presence of the c‐In_2_O_3_ phase in the starting catalyst facilitates its growth at the interface between contacting rh‐In_2_O_3_ particles; thus, the phase transformation occurs very rapidly.^[25]^

The pure rh‐catalyst performs better than the mixed c‐ and rh‐catalysts in terms of STY_MeOH_, selectivity, and stability, reaching the highest yield at 350 °C. The decrease in the reaction rate is most probably caused by the increase in the crystallite size (i.e., sintering), which is hindered in the pure rh‐catalyst and enhanced for those systems with rh→c transition. The absence of c‐nuclei hinders the transformation of the metastable rh→c polymorph, and the crystallite growth. The temperature and the partial pressure of H_2_ should be carefully chosen to maximize the amount of V_O_: these vacancies are generated above 220 °C and maximized above 275 °C, resulting in an expansion of the unit cell at specific concentrations. Simultaneously, it is necessary to limit the temperature, the P_H2_, and the flow rate to hinder the rh→c→In^0^ transformation. In the mixed‐phase catalyst tested at a higher flow rate, the rh→c transition starts at ~230 °C. Limiting the ratio of H_2_ to 1 : 3 : 1 hinders the over‐reduction to In^0^ even up to 400 °C (in situ XRD is not the ideal technique to assess the exact temperature for the appearance of the molten In^0^/in situ XAFS would be better suited). Moreover, limiting the temperature to 275 °C does hinder the crystallite size growth. Supporting the In_2_O_3_ catalyst on ceramic supports that exploit interfacial effects while enhancing stability and yield seems mandatory for the selective synthesis of methanol from CO_2_.

## Conclusions

Catalyst samples composed of different ratios of cubic and rhombohedral In_2_O_3_ have been synthesized using a solvothermal method and tested in an ideally mixed gas phase reactor for CO_2_ hydrogenation to methanol. The pure rhombohedral sample performs the best in terms of STY to methanol over the full temperature range tested. Synchrotron in situ XRD combined with Rietveld refinement allows to assess the stability of both cubic and rhombohedral In_2_O_3_ polymorphs: depending on the temperature, the partial pressure of H_2,_ and the gas flow rate, a rhombohedral to cubic to metallic indium transition occurs. If present, cubic In_2_O_3_ serves as nuclei to induce the rhombohedral to cubic transition, hampering performance.

Upon interaction with H_2_‐containing gas mixtures, the unit cell of both polymorphs expands at the same temperature, indicating the generation of oxygen vacancies. An increase in the partial pressure of H_2_ in the reaction mixture results in a mild decrease of the onset temperature for rh→c transition as well as the generation of V_O_. The current work allows for a rational choice of the polymorph of In_2_O_3_ and defines the operating window to maximize stability and yield. It seems necessary to exploit the combination with ceramic support to exploit interfacial effects and maximize the stability and yield of methanol over the In_2_O_3_ catalyst.

## Author Contributions

Conceptualization was performed by A. Gili, M.F. Bekheet; data curation was done by A. Gili, F. Hess, M.F. Bekheet; formal analysis was performed by A. Gili, G. Brösigke, F. Hess, R. Schomäcker, M.F. Bekheet; funding acquisition was performed by R. Schomäcker, A. Gurlo; Resource provision was done by M. Javed; investigation was done by A.Gili, E. Dal Molin, M. Javed, G. Brösigke, P. Isbrücker, M.F. Bekheet, F. Hess; project administration was performed by A. Gili, M.F. Bekheet; writing, review and editing were done by all co‐authors.

## Conflict of Interests

The authors declare no competing financial interest.

1

## Supporting information

As a service to our authors and readers, this journal provides supporting information supplied by the authors. Such materials are peer reviewed and may be re‐organized for online delivery, but are not copy‐edited or typeset. Technical support issues arising from supporting information (other than missing files) should be addressed to the authors.

Supporting Information

## Data Availability

The data that support the findings of this study are available from the corresponding author upon reasonable request.
