# Supplementary material for: Performance and Stability of Corundum‐type In2O3 Catalyst for Carbon Dioxide Hydrogenation to Methanol
Source: Angew Chem Int Ed Engl. 2025 Jan 10;64(5):e202416990. doi: 10.1002/anie.202416990 (PMC11773125; doi:10.1002/anie.202416990)
Supplement: Supplementary file 1 — Supporting Information [file ANIE-64-e202416990-s001.pdf]

Angewandte Chemie
Eine Zeitschrift der Gesellschaft Deutscher Chemiker

Supporting Information

Performance and Stability of Corundum-type In_2O_3 Catalyst for Carbon Dioxide Hydrogenation to Methanol

A. Gili, G. Brösigke, M. Javed, E. Dal Molin, P. Isbrücker, J.-U. Repke, F. Hess, A. Gurlo, R. Schomäcker, M. F. Bekheet**

Supporting information of

Performance and Stability of Corundum-type In_2O_3 Catalyst for Carbon Dioxide Hydrogenation to Methanol

Albert Gili[1,2,], Georg Brösigke[3], Mudassar Javed[3], Emiliano Dal Molin[4], Philipp Isbrücker[1], Jens-Uwe Repke[3], Franziska Hess[1], Aleksander Gurlo[4], Reinhard Schomäcker[1], Maged F. Bekheet[4,*]*

[1] *Technische Universität Berlin, Faculty II Mathematik und Naturwissenschaften, Institut für Chemie, Straße des 17. Juni 135, 10623 Berlin, Germany*

[2] *Helmholtz-Zentrum Berlin für Materialien und Energie, 14109, Berlin, Germany*

[3] *Process Dynamics and Operations Group, Technische Universität Berlin, KWT9, Straße des 17. Juni 135, 10623 Berlin, Germany*

[4] *Technische Universität Berlin, Faculty III Process Sciences, Institute of Materials Science and Technology, Chair of Advanced Ceramic Materials, Straße des 17. Juni 135, 10623 Berlin, Germany*

*** Corresponding authors:** Albert Gili, albert.gili@helmholtz-berlin.de, Maged F. Bekheet maged.bekheet@ceramics.tu-berlin.de

This supporting information document contains:

Methods description

Figures SI1-SI9

Tables SI1-SI2

Methods

Catalyst preparation. The In_2O_3 catalysts were synthesized using a solvothermal method, as reported elsewhere[1]. In brief, 2.5 g of indium nitrate ($In(NO_3)_3 \cdot xH_2O$, 99.9 %, metal basis, Sigma-Aldrich), was dissolved in 70 ml of anhydrous ethanol solvent (ROTIPURAN, 99.8 %, p.a., Carl Roth) at room temperature under continuous stirring, followed by the dropwise addition of 1.5 M sodium hydroxide (NaOH, pellets, >99 %, p.a., Carl Roth) in ethanol solution until the pH of the solution reached 9. The obtained white precipitate was washed three times with ethanol before being further dispersed in 40 ml of fresh ethanol solvent. The solution's pH value was further increased to 12.75 by the addition of sodium hydroxide before transferring it into a 100 ml Teflon-lined steel autoclave, and solvothermal synthesis was performed at 150 °C for 24-72 hours. After cooling to room temperature, the solvothermal products were separated by centrifugation, washed two times with distilled water, and dried overnight at 60 °C in air, before calcination at 375 °C in air to obtain the nanosized oxides. The phase composition of the samples (*i.e.*, wt.% of rh-In_2O_3:c-In_2O_3) was controlled by controlling the solvothermal time and amount of water H_2O in the solvothermal solvent[1]. The samples used in this study are described as %rh-In_2O_3, being the % of rh extracted from Rietveld refinement of XRD data after synthesis. The commercial Cu/ZnO/Al_2O_3 (CZA, CuO:ZnO:Al_2O_3:MgO= 63.5:25:10:1.5 wt%) catalyst was purchased from Alfa Aesar/Thermo Scientific Chemicals.

Catalytic testing. The catalysts were tested in a lab-scale Berty reactor (1" I.D. Mircro Catalytic Packless Reactor, Autoclave Engineers, US) of 36 mL of volume. A simplified P&ID is depicted in Fig. S1. The setup is described in detail elsewhere[2]. Briefly, the educts are supplied via gas bottles (technical grade, Linde plc, IE) and the gas composition and flow rate are controlled via individual MFCs (EL-FLOW-Prestige (CO_2), EL-FLOW-Select (N_2, H_2), Bronkhorst, NL). The temperature is monitored using a K-type thermocouple (NiCr/NiAl) (TC, TC Mess- und Regeltechnik GmbH, GER) and a ceramic heating sleeve controlled using a PID controller (3216, Eurotherm, GER). The pressure inside the reactor is monitored (Cerabar PMP21, E+H, GER) and controlled using a pressure regulating valve (1/4" G18129 P9, Badgermeter, US). The downstream lines are heated at 180 °C to prevent condensation before analysis. The product stream is analyzed using an online micro gas chromatograph (µGC 490, Agilent, US) equipped with CP-Molsieve 5Å and PoraPLOT Q columns, heated sample lines, and heated injectors. Prior to testing, the catalyst samples were hydraulically pressed, crushed, and sieved to obtain a particle size distribution of 200-400 µm. The samples were diluted with 0.4-0.8 mm quartz particles (Carl Roth GmbH + Co. KG, GER) before being loaded to the reactor at a catalyst-to-diluent ratio of 0.07:1. The turbine of the Berty was set to 2500 rpm. The catalysts were initially heated to 350 °C, using a heating rate of 2 $°C \cdot min^{-1}$ under N_2 flow (WHSV= 20000 $NmL \cdot h^{-1} \cdot g_{cat}^{-1}$), and pressurized to 20 bar (abs) and the conditions were held for 1 hour. Subsequently, the reaction mixture was changed to a CO_2:H_2:N_2= 1:3:1 at WHSV=20000 $NmL \cdot h^{-1} \cdot g_{cat}^{-1}$. This gas composition and flow rate were kept constant during the rest of the experiment. The temperature was decreased in 25 °C intervals down to 250 °C at 1 $°C \cdot min^{-1}$, and later risen to 350 °C at 2 $°C \cdot min^{-1}$ to obtain a measure of deactivation by comparing the first and last data point of a single experiment. The conditions were kept constant until steady state behavior in the product concentrations was observed at each temperature setpoint with a minimum time of 3 hours. The catalysts were recovered for post-catalytic XRD after testing.

Data reconciliation. The raw measured data from the µGC is prone to error, as the mass and mole fractions do not sum up to 1. As there is strong redundancy in the data (8 measured concentrations linked by only 2 reactions) the raw data was reconciled on basis of element balances for N, H, O and C and summation equations at inlet and outlet stream. The Matlab® optimization solver *fmincon* is used with nonlinear constraints g featuring the element balances and the summation equations. The objective function f to be minimized is the sum of the squared differences between measured and calculated value.

Equation 1 $$\min_x f(x) = \sum (x_{meas} - x_{calc})^2$$

Equation 2 $$\textit{subject to: } g(x) = 0$$

Thermodynamic calculations. The change in the Gibbs free energy for the chemical reactions (*i* reaction) was obtained from the Gibbs-Helmholtz equation (*Equation 3*), and the enthalpy and entropy change was calculated using *Equation 4* and *Equation 5*. Different sources of the enthalpies and entropies of formation were used[3,4].

Equation 3 $$\Delta G_i^\circ = \Delta H_i^\circ - T \cdot \Delta S_i^\circ$$

Equation 4 $$\Delta H_i^\circ = \sum \varphi_f \Delta H_{f,prod}^\circ - \sum \varphi_f \Delta H_{f,react}^\circ$$

Equation 5 $$\Delta S_i^\circ = \sum \varphi_f \Delta S_{f,prod}^\circ - \sum \varphi_f \Delta S_{f,react}^\circ$$

DFT calculations. The density functional theory (DFT) calculations were performed using the Vienna Ab-initio Simulation Package (VASP)[5] version 6.4.1 using the Perdew-Burke-Ernzerhof (PBE) functional[6] including Grimme's D3 dispersion correction[7]. We employed the default "In" and "O" pseudopotentials. V_O in c- and rh-In_2O_3 were computed in supercells containing 32 and 48 $InO_{1.5}$ units, respectively, resulting in defect concentrations between 0 and 7%. The resulting structures in CONTCAR format in the supplementary material, including a tabular overview of the lattice expansion and oxygen vacancy formation energies. All super cells were calculated at a constant k-point density in reciprocal space at 72 k-points/Å^{-1}. An energy cutoff of 550 eV was employed. Lattice parameters and ion positions were simultaneously relaxed, and the convergence criteria for the energy is set to 10^{-6} eV during structural optimization.

Defect calculations and stability. To approximate the maximum V_O concentration under reaction conditions, we calculated the V_O formation energy (ΔE_V) based on the defect formation equation in Kröger-Vink notation:

$$O_O^x + H_2 \rightarrow V_O^{\cdot\cdot} + 2e' + H_2O$$

This results in 1.01 eV and 1.14 eV for the c- and rh-polymorphs at a concentration of 1%, which was obtained by fitting the available data points (Figure SI7). Employing phonon calculations in the phonopy package using the finite differences method with a displacement of 0.01 Å, we calculated the lattice entropies of the stoichiometric structures, resulting in 106.2 J· K^{-1}·mol^{-1} and 101.3 J· K^{-1}·mol^{-1}for the rhombohedral and cubic polymorphs, respectively. The higher entropy of the rhombohedral polymorph suggests that this modification may be more stable than the cubic at higher temperature; however, in the studied temperature range up to 350 °C, the formation energy is the decisive factor governing the stability. Figure S19 shows the formation energies with reference to In metal and gaseous O_2, for defective In_2O_3 polymorphs, as a function of the oxygen vacancy concentration. It is evident that, in terms of energy, the cubic polymorph is more stable than the rhombohedral, by 65.7 meV on average per InO_x unit. Oxygen vacancies have a negligible influence on the relative stabilities of the two polymorphs. Based on the large energy difference and small entropy difference between the two polymorphs, we expect that lattice entropy effects in this system will not have a significant influence at temperatures lower than 1300 K. In the temperature range studied here, lattice energy is the decisive factor governing the relative stabilities of cubic and rhombohedral polymorphs.

Lattice expansion. Cell volumes as a function of defect concentrations were obtained by relaxing the lattice parameters in different super cells up to 64 In_2O_3 units with variable number of missing oxygen atoms. Figure SI8 shows the resulting relative lattice expansion for the rh- and c-polymorphs. For both, a lattice expansion is observed at low concentration, while shrinkage sets in at higher concentration. In the case of the rh-structure, the lattice expansion is maximized at 0.7% V_O, where the lattice is expanded by 0.17%. In the c-structure, the maximum is found between 2.1% V_O, with 0.9% expansion.

Catalyst characterization.

The specific surface area (SSA) was quantified by nitrogen sorption in two different setups. Firstly, a QuadraSorb Station 4 apparatus (Quantachrome, USA): the isotherms were obtained at 77 K after degassing for 12 hours at 200 °C (under vacuum). The Quantachrome/QuadraWin software version 5.05 was used to analyze all nitrogen sorption data. Brunauer−Emmett−Teller (BET) calculations were applied to obtain SSA. Secondly, a BELSORF Mini II from MicrotracBEL (Japan), after degassing at 300 °C under vacuum overnight.

X-ray diffraction (XRD) was performed in 2 different setups. *Ex-situ* XRD was performed using a Bruker D8 diffractometer (Bruker AXS GmbH, Germany) operating with a Cu-$K_{\alpha1}$ radiation source (1.5406 Å). The measurements were performed using a Bragg-Brentano geometry between 10° and 20° angles, with a step size of 0.02° and 1 second per step. The instrument was calibrated using a LaB_6 NIST 660b standard. *In-situ* X-ray diffraction (XRD) was performed in the P02.1 beamline of the PETRA III synchrotron in DESY, Hamburg, Germany[8,9]. The setup and cell were designed and constructed by our group and are described in detail elsewhere[10]. Briefly, 1-2 mg of powder catalyst are loaded in between silica wool beds inside an amorphous quartz capillary of 1 mm internal diameter (Hilgenberg, Germany) that acts as plug-flow packed bed reactor/cell while being inert and mostly transparent to X-rays. A SiC tube (kindly provided by Fraunhofer IKTS, Germany) concentrical to the quartz capillary acts as a furnace by being illuminated by two 64635 HLX IR lamps (Osram, Germany). A k-type thermocouple (Reckmann, Germany) is placed next to the catalyst sample, downstream to prevent altering the gas composition before interacting with the sample. This TC serves to control the cell temperature by a PID controller programmed with Labview software. 3 El-Prestige mass flow controllers (MFC, Bronkhorst, The Netherlands) precisely control the flow rate and gas phase composition. A pressure indicator (Omega, UK) allows monitoring of the pressure close to the sample position, which is regulated by the pressure regulation unit, composed of an El-Press pressure regulator (Bronkhorst, The Netherlands) and an Equilibar research series back-pressure regulator (Equilibar, USA). All the instruments are controlled and data is logged using a single Labview program. The setup is operated from the control room of the beamline. All experiments were performed using an X-ray wavelength of 0.2073 Å (60 keV). The 2D images were collected using a Varex XRD 4343CT detector (150x150 μm^2 pixel size), with 30 s acquisition time, utilizing a 0.5x0.5 mm^2 beam size. The sample-detector distance and instrumental peak broadening were calibrated with a LaB_6 NIST 660b standard. The 2D images were integrated using Dioptas[11]. Besides the *in-situ* data, *ex-situ* patterns were obtained in the same beamline by loading small amounts of the powder catalysts inside quartz capillaries and rotating them. The XRD data was refined using Rietveld refinement, which was performed with FULLPROF software[12] with profile function 7 (Thompson-Cox-Hastings pseudo-Voigt convoluted with an axial divergence asymmetry function)[13]. The structure refinement of a LaB_6 standard allowed us to determine the resolution function of the instrument. The absolute errors for the determination of the wt.% are often ±0.6 and always below ±1; often $\pm 2 \cdot 10^{-4}$ for the cell parameters (propagation of errors has been applied to calculate the errors in the cell volume/Z); the errors for the crystallite size are always below ±1. The data was further processed using Origin software[14].

Table SI1. Wt.% of rhombohedral rh-In_2O_3 (R-3c, PDF #00-022-0336) and cubic c-In_2O_3 (Ia-3, PDF #00-006-0416) polymorphs obtained with refinement of the ex-situ XRD data as well as SSA area obtained using BET-N_2 adsorption.

Sample	Fresh catalysts				Spent catalysts				Surface area [$m^2 \cdot g^{-1}$]
	Weight fraction [wt%] ±1.0		Crystallite size [nm] ±1.0		Weight fraction [wt%] ±1.0		Crystallite size [nm] ±1.0		
	rh-In_2O_3	c-In_2O_3	rh-In_2O_3	c-In_2O_3	rh-In_2O_3	c-In_2O_3	rh-In_2O_3	c-In_2O_3	
100% rh-In_2O_3	100	0	9.5	-	100	0	13.9	-	41.0
80% rh-In_2O_3	80	20	15.0	17.9	62	38	22.0	25.3	26.0
58% rh-In_2O_3	58	42	11.4	13.5	20	80	21.8	18.8	38.0
Cu/ZnO/ Al_2O_3	-	-	-	-	-	-	-	-	101.0

Table SI2. Crystallographic data of the different In-containing compounds detected.

Formula	PDF	Space group	Cell parameters [Å]	Cell volume [Å^3]	Z
In_2O_3	00-006-0416	Ia-3	a= 10.118	1035.82	16
In_2O_3	00-022-0336	R-3c	a= 5.487<br>c= 14.51	378.33	6
In	00-005-0642	I4/mmm	a= 3.252<br>c= 4.946	52.3	2

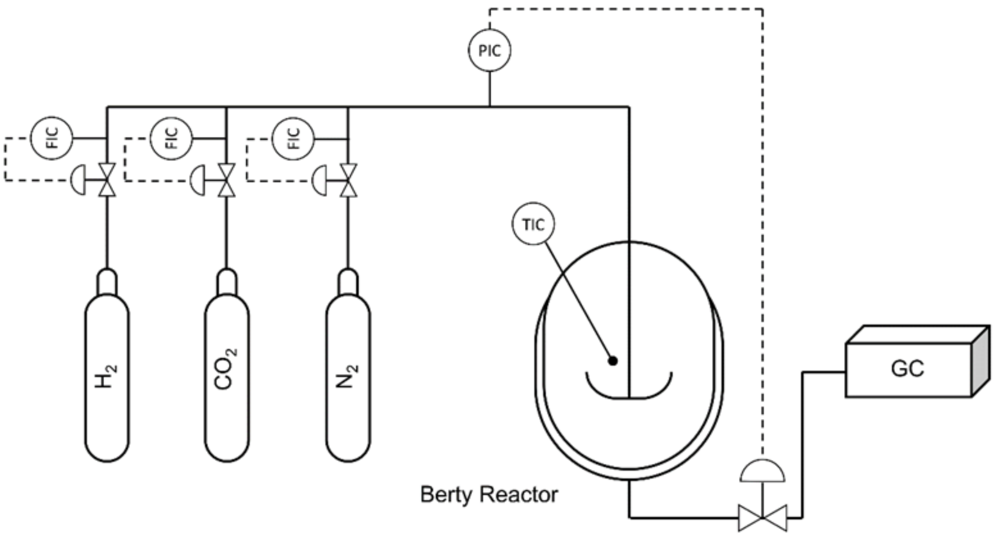

Figure SI1. Simplified P&ID of the testing facility with a Berty reactor and µGC.

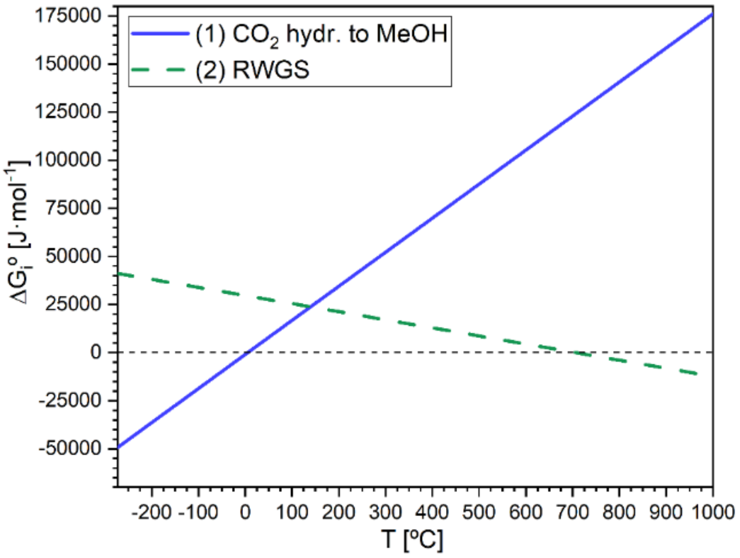

Figure SI2. $\Delta G_i°$ for reactions (1), $3H_2 + CO_2 \leftrightarrow CH_3OH + H_2O$ and (2) $H_2 + CO_2 \leftrightarrow CO + H_2O$ as function of the temperature.

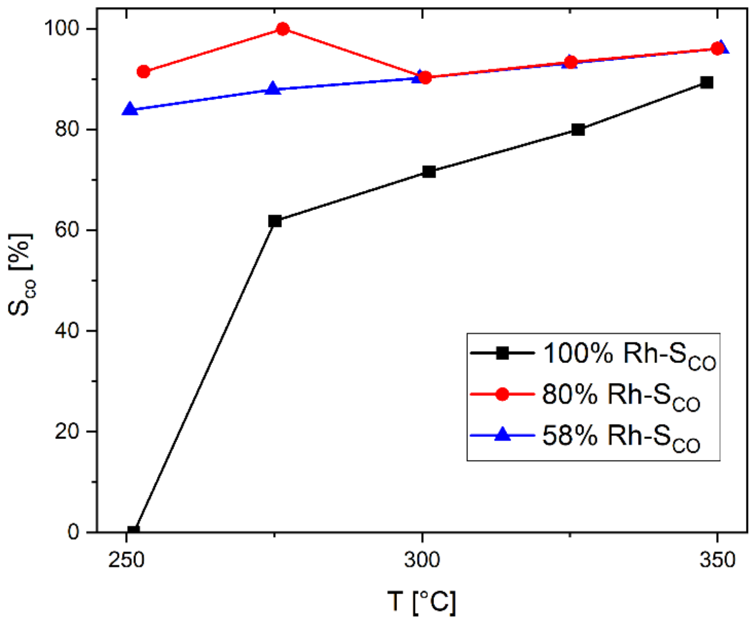

Figure SI3. Selectivity toward CO of all three samples as function of the temperature, same experiment as shown in figure 1 of the main text.

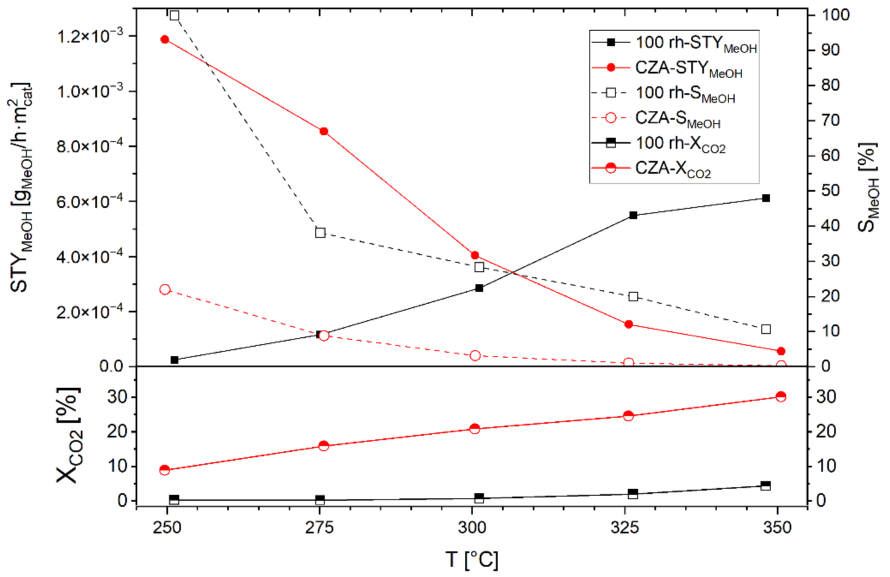

Figure SI4. (Top) STY_{MeOH} and S_{MeOH} and (bottom) X_{CO2} as a function of the temperature for the 100% rh-In_2O_3 and the commercial CZA ($Cu/ZnO/Al_2O_3$) catalysts. Conditions: P= 20 bar, WHSV= 20000 $NmL \cdot h^{-1} \cdot g_{cat}^{-1}$, CO_2:H_2:N_2= 1:3:1 [-]. Lines are added for easier visualization.

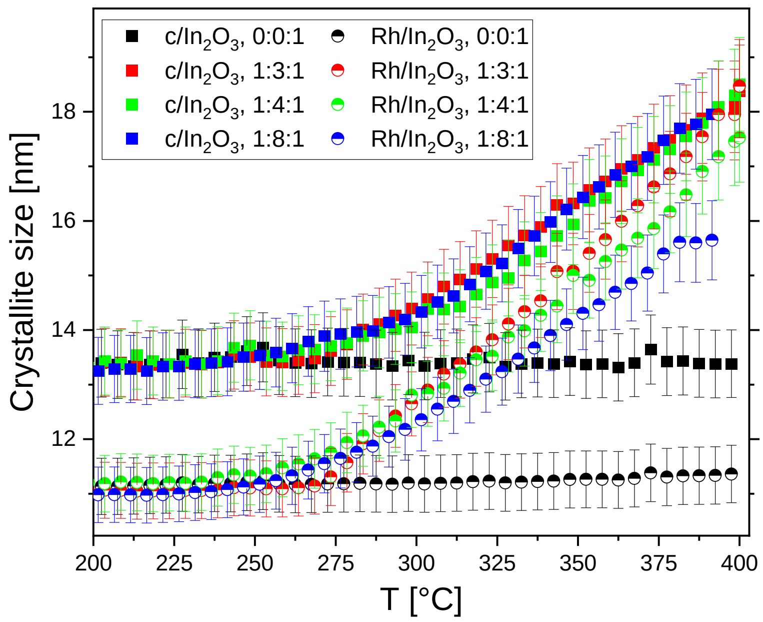

Figure SI5. Crystallite size obtained from refinement of the in-situ data shown in Figure 2 (58%rh/In_2O_3 sample) during the heating step. Conditions: P= 20 bar$_{(abs)}$, total flow rate of 5 NmL·min^{-1} for reaction (WHSV~ 200000 NmL·h^{-1}·g$_{cat}$$^{-1}$) heating rate is 10 °C·min^{-1}.

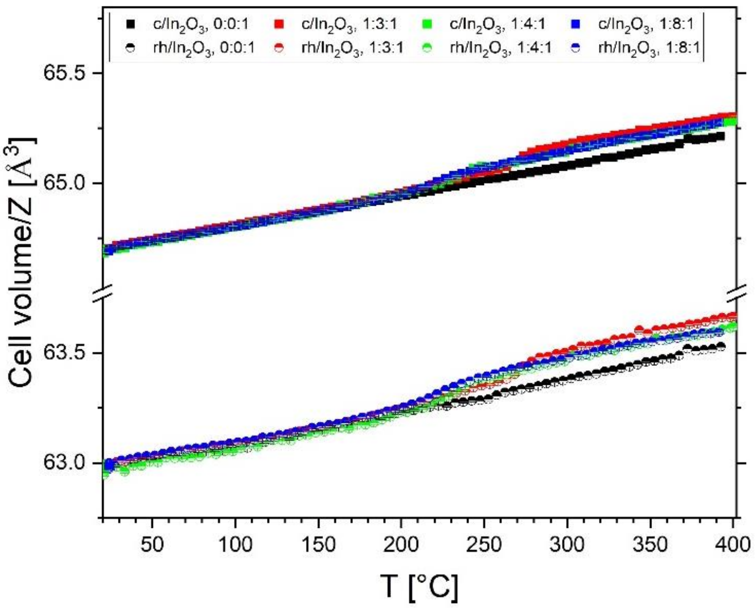

Figure SI6. Cell volume/Z for the heating step of the 58%rh/In_2O_3 sample obtained from refinement of the in-situ data shown in Figure 2. Conditions: P= 20 bar$_{(abs)}$, total flow rate of 5 NmL·min^{-1} for reaction (WHSV~ 200000 NmL·h^{-1}·g$_{cat}$$^{-1}$) heating rate is 10 °C·min^{-1}. Magnified figures of the interesting segments are shown in Figure 3 of the manuscript.

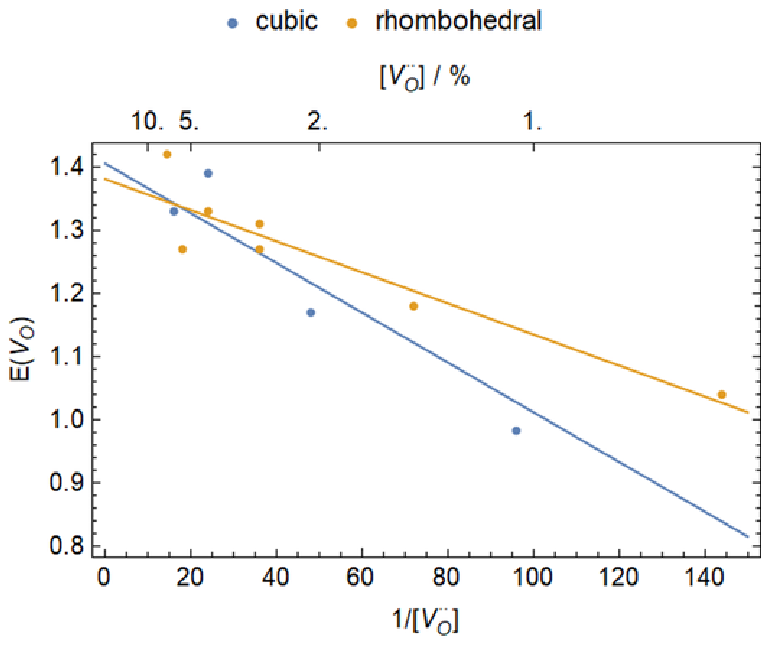

Figure SI7. Oxygen vacancy formation energy for the cubic and rhombohedral polymorphs of In_2O_3. Lines represent a linear regression.

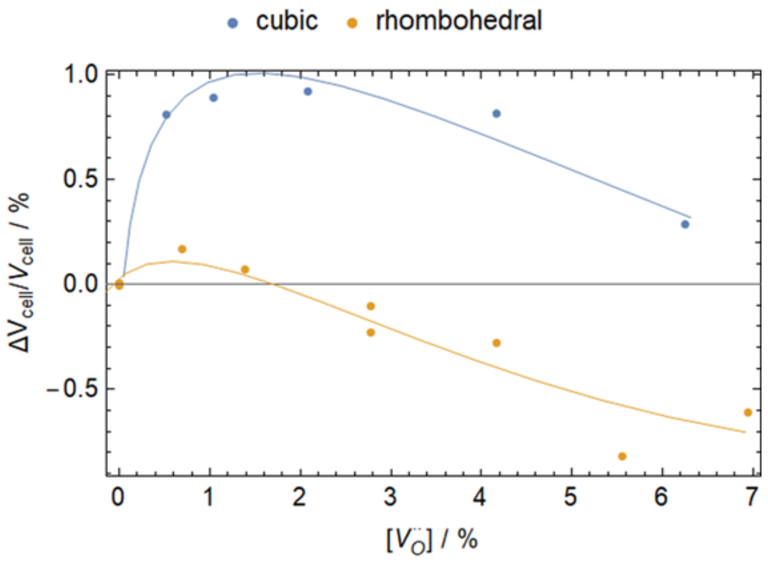

Figure SI8. Volume expansion calculated by DFT as a function of oxygen vacancy concentration for the cubic and rhombohedral polymorphs of In_2O_3. Lines are added for guidance of the eye.

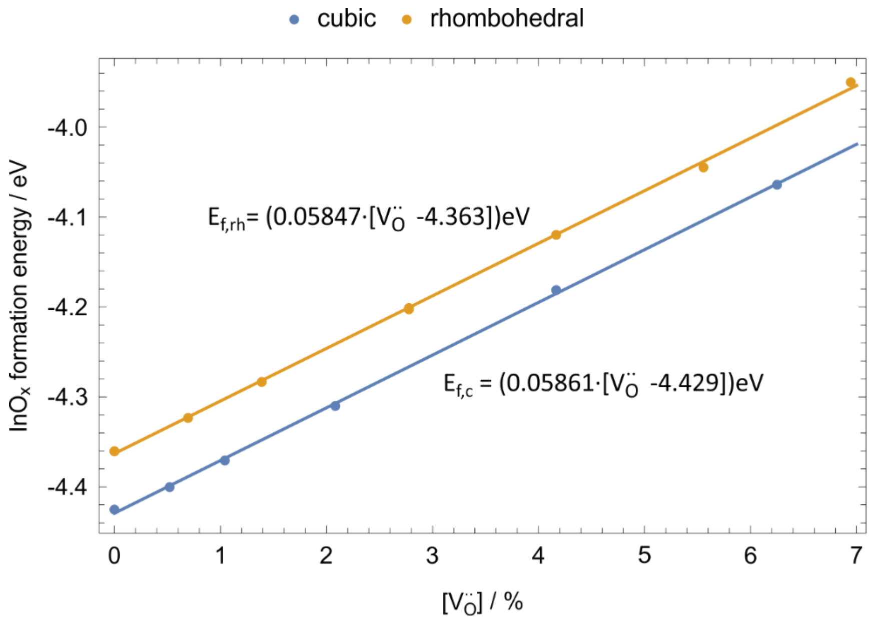

Figure S19. DFT-calculated formation energies for the two In_2O_3 polymorphs as a function of oxygen vacancy concentration. Fitted functions are given in the figure.

References

(1) Schlicker, L.; Bekheet, M. F.; Gurlo, A. Scaled-up solvothermal synthesis of nanosized metastable indium oxyhydroxide (InOOH) and corundum-type rhombohedral indium oxide (rh-In_2O_3). *Z. Kristallogr.* **2017**, *232* (1-3), 322. DOI: 10.1515/zkri-2016-1967.

(2) Javed, M.; Brösigke, G.; Schomäcker, R.; Repke, J.-U. Influence of the Distance between Two Catalysts for CO_2 to Dimethyl Ether Tandem Reaction. *Chem Eng & Technol* **2023**, *46* (6), 1163–1169. DOI: 10.1002/ceat.202200541.

(3) Lide, D. R. *CRC Handbook of Chemistry and Physics, Internet Version 2005*; CRC Press, 2005.

(4) https://www.chemeo.com.

(5) Kresse, G.; Furthmüller, J. Efficient iterative schemes for ab initio total-energy calculations using a plane-wave basis set. *Phys. Rev. B* **1996**, *54* (16), 11169–11186.

(6) Perdew, J. P.; Chevary, J. A.; Vosko, S. H.; Jackson, K. A.; Pederson; Singh, D. J.; Fiolhais, C. Atoms, molecules, solids, and surfaces: Applications of the generalized gradient approximation for exchange and correlation. *Phys. Rev. B* **1992**, *46* (11), 6671–6687. DOI: 10.1103/physrevb.46.6671.

(7) Grimme, S.; Antony, J.; Ehrlich, S.; Krieg, H. A consistent and accurate ab initio parametrization of density functional dispersion correction (DFT-D) for the 94 elements H-Pu. *J. Chem. Phys.* **2010**, *132* (15), 154104. DOI: 10.1063/1.3382344.

(8) Dippel, A.-C.; Liermann, H.-P.; Delitz, J. T.; Walter, P.; Schulte-Schrepping, H.; Seeck, O. H.; Franz, H. Beamline P02.1 at PETRA III for high-resolution and high-energy powder diffraction. *J. Synchrotron Radiat.* **2015**, *22* (3), 675–687. DOI: 10.1107/S1600577515002222.

(9) Schökel, A.; Etter, M.; Berghäuser, A.; Horst, A.; Lindackers, D.; Whittle, T. A.; Schmid, S.; Acosta, M.; Knapp, M.; Ehrenberg, H.; Hinterstein, M. Multi-analyser detector (MAD) for high-resolution and high-energy powder X-ray diffraction. *Journal of synchrotron radiation* **2021**, *28* (Pt 1), 146–157. DOI: 10.1107/S1600577520013223.

(10) Bischoff, B.; Bekheet, M. F.; Dal Molin, E.; Praetz, S.; Kanngießer, B.; Schomäcker, R.; Etter, M.; Jeppesen, H. S.; Tayal, A.; Gurlo, A.; Gili, A. *In situ/operando* plug-flow fixed-bed cell for synchrotron PXRD and XAFS investigations at high temperature, pressure, controlled gas atmosphere and ultra-fast heating. *J. Synchrotron Rad.* **2024** (31). DOI: 10.1107/S1600577523009591.

(11) Prescher, C.; Prakapenka, V. B. DIOPTAS : a program for reduction of two-dimensional X-ray diffraction data and data exploration. *High Pressure Res.* **2015**, *35* (3), 223–230. DOI: 10.1080/08957959.2015.1059835.

(12) J. Rodriguez-Carvajal. Fullprof: A Program for Rietveld Refinement and Pattern Matching Analysis. *Abstract of the Satellite Meeting on Powder Diffraction of the XV Congress of the IUCr, Toulouse, France* **1990**, 127.

(13) Finger, L. W.; Cox, D. E.; Jephcoat, A. P. A correction for powder diffraction peak asymmetry due to axial divergence. *J. Appl. Crystallogr.* **1994**, *27* (6), 892–900.

(14) Origin(Pro), Version 2022b. OriginLab Corporation, Northampton, MA, USA.
